# A Modified Approach for Ultrasound-Guided Thoracic Paravertebral Block via Thoracic Intervertebral Foramen in an Adolescent Patient: A Case Report

**DOI:** 10.3390/jcm11092646

**Published:** 2022-05-08

**Authors:** Emiliano Petrucci, Franco Marinangeli, Barbara Pizzi, Francesco Sciorio, Gioele Marrocco, Massimo Antonio Innamorato, Marco Cascella, Alessandro Vittori

**Affiliations:** 1 Department of Anesthesia and Intensive Care Unit, San Salvatore Academic Hospital of L’Aquila, Via Vetoio 48, 67100 L’Aquila, Italy; 2 Department of Anesthesiology, Intensive Care and Pain Treatment, University of L’Aquila, Piazzale Salvatore Tommasi 1, Coppito, 67100 L’Aquila, Italy; francomarinangeli@gmail.com (F.M.); francesco.sciorio@gmail.com (F.S.); gioelemarrocco9@gmail.com (G.M.); 3 Department of Anesthesia and Intensive Care Unit, SS Filippo and Nicola Academic Hospital of Avezzano, Avezzano, 67051 L’Aquila, Italy; bpizzi@hotmail.it; 4 Department of Neuroscience, Pain Unit, Santa Maria delle Croci Hospital, AUSL Romagna, Viale Vincenzo Randi 5, 48121 Ravenna, Italy; massimo.innamorato@auslromagna.it; 5 Department of Anesthesia and Critical Care, Istituto Nazionale Tumori—IRCCS, Fondazione Pascale, Via Mariano Semmola 53, 80131 Naples, Italy; m.cascella@istitutotumori.na.it; 6 Department of Anesthesia and Critical Care, ARCO ROMA, Ospedale Pediatrico Bambino Gesù IRCCS, Piazza S. Onofrio 4, 00165 Rome, Italy; alexvittori@libero.it

**Keywords:** anesthesia, regional anesthesia, pediatric anesthesia, adolescent, paravertebral block, epidural anesthesia, pain, postoperative pain, anesthetic absorption, local anesthetic

## Abstract

This case report describes a modified approach for a thoracic paravertebral block by performing a bilateral ultrasound-assisted injection of 12 mL of 0.5% levobupivacaine near the thoracic intervertebral foramen, combined with general anesthesia, in a patient who underwent emergent laparotomy for small intestinal volvulus. Two continuous catheter sets were used for a bilateral continuous block with levobupivacaine 0.25% at a rate of 5–8 mL/h. No complications during the execution of the block were recorded. No supplemental opioids were administered and the patient was hemodynamically stable, requiring no pharmacological cardiovascular support during surgery. At the end of the surgical procedure, the patient received a continuous flow of 0.2% levobupivacaine as postoperative analgesia, at a basal flow of 4 mL/h per each side, a bolus of 4 mL, and a lockout time of 60 min was used. The postoperative pain on the Numeric Rating Scale was 2 at rest and it was 4 in motion, without neurological or respiratory sequelae due to block in the first 72 h after surgery.

## 1. Introduction

Regional anesthetic techniques provide anesthesia during surgery followed by effective long-acting postoperative analgesia [1,2]. International literature underlines the role of regional anesthesia in the improvement of perioperative pain control, reducing the request for opioids and related side effects [3,4]. Epidural analgesia (EA) and thoracic paravertebral block (TPVB) are considered useful techniques for the control of postoperative pain of thoracic and visceral abdominal surgery, as well as from the opioid sparing point of view [5]. The current literature on ultrasound-guided TPVB describes a wide range of techniques for use on patients and cadavers, but it is currently not possible to provide an evidence-based recommendation on the choice between techniques. Many factors can influence the weighing of procedure choice, because of individual preferences per individual physician, skills, and experience with other ultrasound-guided regional anesthesia techniques and with landmark-guided blockades [6]. Thus, we hypothesized an alternative way to perform this block, attempting to minimize the relative disadvantages of the various described techniques and the risk of iatrogenic damage [7,8].

During an alternative approach for the TPVB, the local anesthetic (LA) spreads into the thoracic paravertebral space through the needle tip, which is placed over and behind the transverse process (TP) of vertebra, via the thoracic intervertebral foramen (TIF). We found that injecting into this site was successful in clinical terms. This technique was named the TIF block.

Based on our clinical observations and virtual dissections, we hypothesized that injection at this site would result in an effective injection spread, presumably because the thoracic paravertebral space (TPVS) and epidural space (ES) could be reached by TIF [9].

In this case report, we performed a bilateral continuous TIF block, providing opioid-free anesthesia and postoperative analgesia for emergent laparotomy (EL) due to a small intestinal volvulus.

## 2. Case Presentation

On 12 October 2021, at the San Salvatore Academic Hospital of L’Aquila (Italy), the alternative approach to thoracic paravertebral block (TPVB) was performed on a 17-year-old male who had EL for a small intestinal volvulus associated with bowel obstruction and ischemia. The patient was 165 cm tall and weighed 70 kg (BMI was 25.71). He was taking bronchodilators and inhaled corticosteroids because of asthma. The American Society of Anesthesiologists (ASA) status was II. A consent form, signed by a legal surrogate of the patient, was obtained for the execution of the anesthesia, while the consent for publication was subsequently signed by the patient, who, in the meantime, had reached the age of majority according to Italian law.

**Figure 1:**
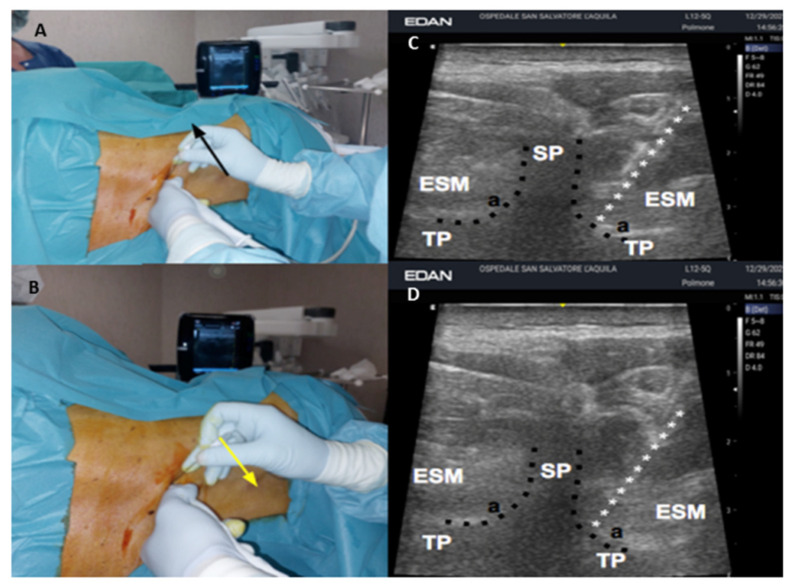
Ultrasound-assisted thoracic intervertebral block. The anesthesiologist injected local anesthetic by overcoming the angle (a, black points) between the spinous (SP) and transverse process (TP) of vertebra. (**A**) The needle back was moved upwards (black arrow), directing the tip from the cephalic to caudal position; (**B**) the needle back was moved down (yellow arrow), directing the tip from the caudal to cephalic position. The transducer was slightly moved from the medial to lateral direction, while maintaining a transverse orientation and observing the angle (a) between SP and TP. This was visualized as a caved structure (black points) that lay deep on the fascial plane of the erector spinae muscle (ESM). The needle (white stars) was inserted and advanced along the inferior (**C**) and the superior (**D**) limit of the angle between TP and SP, until losing contact with the bone.

**Figure 2:**
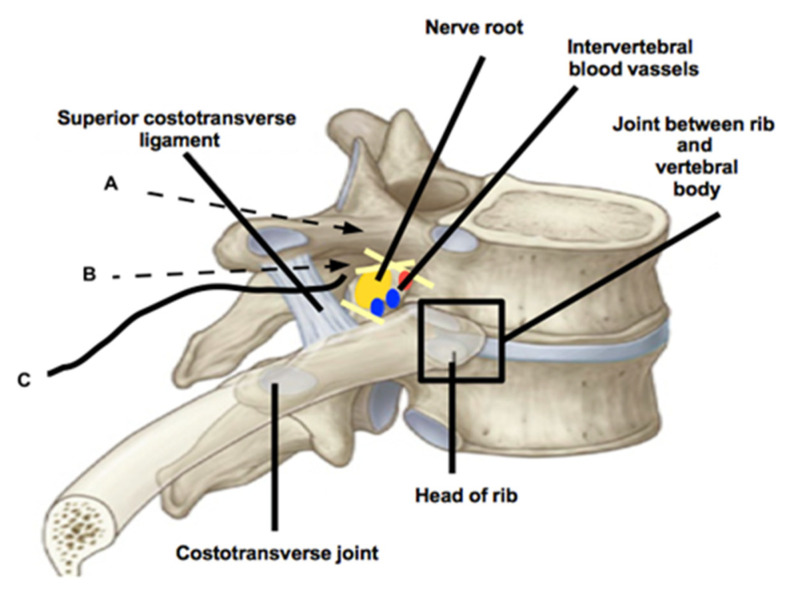
Needle direction and catheter position. Black arrow A indicates the needle tip position in the cephalic to caudal direction; black arrow B indicates the needle tip in the caudal to cephalic direction. Black line C is the catheter position, inserted from the caudal to the cephalic direction. Yellow lines represent the transforaminal ligaments.

Peripheral venous access was obtained and 2.5 mg of midazolam and 0.1 mcg·kg^−1^ sufentanil were administered intravenously before execution of the block. The patient was placed in the left and then right lateral position to perform the bilateral block. Ultrasonography was performed from the seventh cervical spinous process (SP) to the tenth thoracic SP vertebra. The tip of the spinous process (SP) of the tenth thoracic vertebra (T_10_) was identified using a high-frequency linear array ultrasound (US) transducer (EDAN, Acclarix AX4, Rome, Italy), which was transversally placed. We began ultrasound scanning in the transverse plane, visualizing the tip of spinous processes as hyperechoic, round shapes with acoustic shadowing beneath it. A protective plastic sheath was used for the US procedure. The transducer was slightly moved from the medial to the lateral direction, while maintaining a transverse orientation and was observed at the angle between the SP and TP. This was visualized as a caved structure that lay deep on the fascial plane of the erector spinae muscle (ESM). The skin site of injection was anesthetized with 2 mL of lidocaine 1%. A Tuohy needle (18 gauge, 90 mm, Contiplex, BBraun, Bethlehem, PA, USA) was gently inserted, in-plane to the US beam in a lateral-to-medial direction to contact the SP, into the skeletal muscle plane of the erector spinae muscle (ESM). Then, the needle tip was moved from the cephalic to the caudal direction, tilting the probe in the same direction when the angle between the TP and SP was reached (Figure 1). Subsequently, the needle tip was gently inserted and advanced 2 mm along the superior limit of the vertebral pedicle, until losing contact with the bone. Six milliliters of levobupivacaine 0.5% were subsequently injected. Similarly, the same anesthetic solution was injected from the caudal in the cephalic direction, overcoming the inferior articular process, 2 mm along the inferior limit of the vertebral pedicle (Figure 1 and Figure 2). Injection pressure monitoring was provided by using the half-the-air technique through a three-way stopcock, which was used to keep the injection pressure below 15 psi [10,11]. The anesthetic procedure was performed bilaterally. Two continuous catheter sets were used and threaded 1 cm from the needle tip for a bilateral continuous block. The catheters were inserted from the caudal in the cephalic direction (Figure 2) and were secured using a cyanoacrylate tissue adhesive (Dermabond, Ethicon, Somerville, NJ, USA) and two layers of a transparent adhesive dressing (TegadermTM, 3M, Maplewood, MN, USA) to prevent retrograde leakage. Cold tests and touch tests were performed bilaterally every 2 min and the patient was judged operable when a loss of cold and touch sensations was observed for the T_7_ to T_12_ dermatomes, in a 4 cm lateral line to the thoracic spine and to the parasternal line.

General anesthesia was induced with propofol 2 mg·kg^−1^. Rocuronium 0.6 mg·kg^−1^ was administered to facilitate the intubation with an endotracheal tube. Anesthesia was maintained with sevoflurane 1.5–2% in oxygen with positive pressure ventilation in a circle system. A bolus of 5 mL levobupivacaine 0.5% was injected through the catheter, followed by a continuous infusion of levobupivacaine 0.25% at a rate of 5–8 mL/h (2.5–4 mL/h for side, titrated to patient weight and clinical effect) using an infusion pump (CADD-Solis, Smiths Medical, Dublin, OH, USA), before surgical incision. ASA guidelines for anesthetic monitoring were respected [12].

No complications due to the execution of the block were recorded. No supplemental opioids were administered and the patient was hemodynamically stable, requiring no pharmacological cardiovascular support during surgery. At the end of surgical procedure, the patient was admitted into the post-anesthesia care unit (PACU) phase 1 and then into the PACU phase 2, before going to the ward [13]. The patient received a continuous flow of 0.2% levobupivacaine as postoperative analgesia, with a basal flow of 8 mL/h (4 mL/h for side), bolus of 4 mL, and a lockout time of 60 min. No pharmacological cardiovascular support was required to the patient’s hemodynamic stability during his stay in the PACU. Acute pain at rest (on laying position), and in motion (during a deep breath) were recorded at 36, 48, and 72 h after surgery. The Numeric Rating Scale for pain (NRS, an 11-point numeric scale, from “0” (“no pain”) to “10” (“worst pain imaginable”)) was used. No patient discomfort, infections, side effects of local anesthetic (LA), nor other complications were observed. Postoperative pain was 2 at rest, and it was 4 in motion, without neurological or respiratory sequelae in the first 72 h after surgery due to execution of the block. Bowel function recovery was recorded after 8 h from surgery. The infusion of analgesic solution was interrupted 76 h after surgery and the catheters were removed. The patient required a mean of 4 bolus per day administered using a PCA pump. Acetaminophen 2000 mg per day was systematically administered in the first 96 h after surgery, without the use of supplemental opioids or non-steroidal anti-inflammatory drugs.

**Figure 3:**
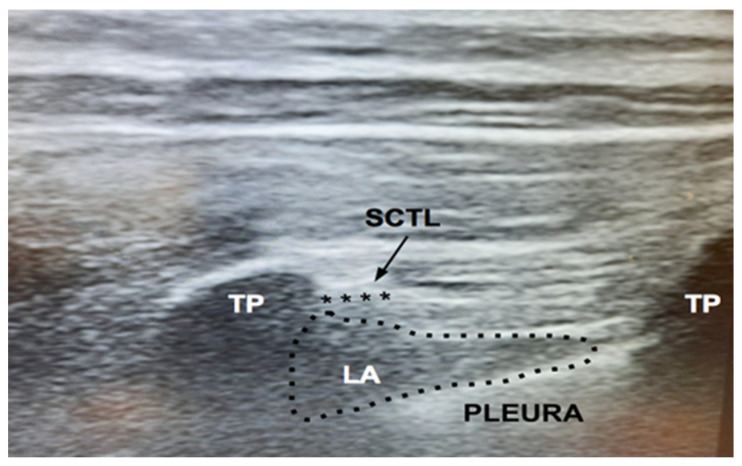
Second-look ultrasound scan. The transducer was placed in the sagittal plane, between transverse processes (TPs) of the eighth (T_8_) and ninth (T_9_) vertebra. Anechoic fluid in the thoracic paravertebral space (TPVS, black points), (T_8_) presumably indicating the local anesthetic (LA) spread. TP, transverse process of T_8_ and T_9_. The TPVS was located between the hyperechoic lines of the superior costotransverse ligament (SCTL, black arrow and stars) and the pleura.

Before catheter removal, a second-look ultrasound scan of thoracic paravertebral space (TPVS) was performed, documenting anechoic fluid in the TPVS at a level of T_8_ and presumably indicating the LA spread (Figure 3).

## 3. Discussion

Epidural anesthesia (EA) and thoracic paravertebral block (TPVB) are considered useful for anesthesia and postoperative analgesia in thoracic and visceral abdominal surgery, achieving useful pain relief and avoiding the side effects of opioids. However, it should be emphasized that these regional anesthesia techniques can be burdened by possible intrinsic side effects, especially in the pediatric population, which has some important peculiarities [7,8,14]. This case description demonstrates that an injection point 2 mm over and behind the angle between the TP and SP achieved spread of dye into the thoracic paravertebral space (TVPS) via thoracic intervertebral foramen (TIF) at the level of injection, and to adjacent levels, providing thoracic and visceral abdominal anesthesia and analgesia.

We performed this anesthetic procedure, presuming that it was an alternative approach for TPVB. Surprisingly, we believe to have found an alternative way to also perform EA under US assistance.

Recently, the erector spinae plane block has been described as more effective at reducing postoperative opioid consumption and pain scores for a broad spectrum of surgeries involving incisions from T_1_ to L_4_ [15]. The hypothesis is that, by injecting anesthetics in this block, they may centrally reach the ventral and posterior branches of the thoracic and thoracolumbar spinal nerves, communicating branches, and sympathetic trunk, and also spread into the ES [16].

**Figure 4:**
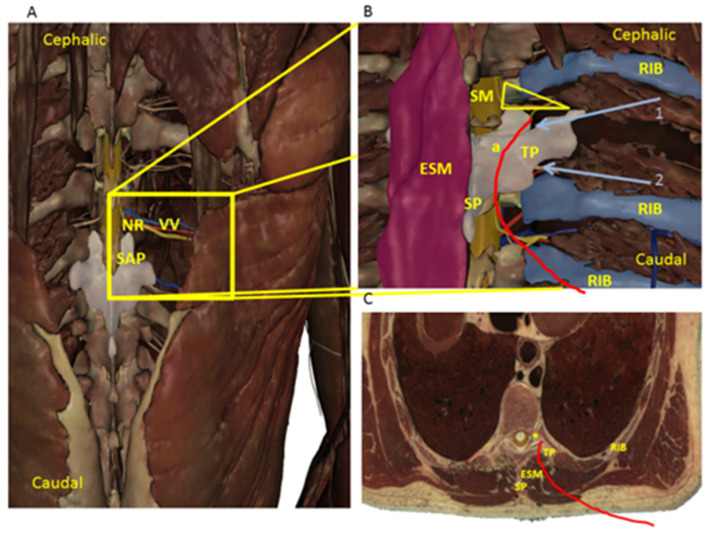
Virtual dissection. (**A**) Virtual dissection of thorax: posterior wall; skin, subcutaneous tissue, and all muscle plane were dissected, reaching the spine. Eighth and ninth vertebrae were removed to identify nerve root (NR) and intervertebral blood vessels (VV). The tenth thoracic vertebra is represented in white. SAP: superior articular process. (**B**) The red line identifies the catheter position inserted in the caudal to the cephalic direction, outside the boundaries of the thoracic paravertebral space (yellow triangle). a, angle between spinous (SP) and transverse process (TP). 1. Light blue arrow indicates the needle tip position from the cephalic to the caudal direction; 2. light blue arrow indicates the needle tip in the caudal to the cephalic direction. (**C**) Transverse plane virtual dissection: the red line identifies the catheter position lying on the ESM plane, over and behind the TP, from the angle between SP and TP, close to the thoracic intervertebral foramen (yellow star).

Although the anatomical boundaries of the TPVS are well described, there is still controversy regarding the injection point of a local anesthetic (LA) because of the complex thoracic paravertebral anatomy. This indeed underlines the hypothesis of the anatomical space of the TPVS. The TPVS is commonly described as triangular-shaped, located bilaterally alongside the whole length of the thoracic vertebral column, filled with fat, and traversed by the dorsal branches and ventral branches of spinal nerves, communicating branches, intercostal nerves and blood vessels, hemiazygos vein, thoracic duct, and sympathetic trunk [17]. The vertebral column forms the base, and the intercostal space is the apex of the TPVS. The parietal pleura represents the anterolateral boundary and the transverse processes of the vertebrae; the head and neck of the ribs form the posterior boundary. The psoas muscle at L_1_ is considered the caudal boundary of the TPVS but the cranial boundary remains undescribed [17]. It is important to underline that, medially, the TIF were found as TPVS boundaries. The TPVS appeared to communicate with the ES and with the contralateral paravertebral space through the intervertebral foramen (IVF) [17]. The TIF is an oval area, laterally faced. Medially, there is the dural sleeve with its emerging nerve root. Laterally, there is a fascial sheet that is part of the anterior layer of the thoracolumbar fascia. Usually, there are two separate oval perforations in this fascia: a posterior perforation for the nerve root (“*nerve root compartment*”) and a smaller anterior perforation for the intervertebral blood vessels (“blood vessels compartment”) [18]. Various foraminal ligaments limit the posterior compartment of the fascia, closely related to the exiting nerve root. Anteriorly, the superior corporopedicular ligament extends from the superior pedicle traversing to the posterolateral vertebral body. Superiorly, the superior transforaminal ligament extends from the arches of the superior and inferior vertebral notches to the articular capsule of the superior pedicle. Inferiorly, the mid-transforaminal ligament runs from the annulus fibrosus and superior and inferior corporopedicular ligaments to the articular capsule. The inferior transforaminal ligament extends from the junction of the annulus fibrosus and the posterior vertebral body to the superior articular facet, limiting the nerve to the vascular compartment (Figure 2 and Figure 4). The number of dorsal rootlets that emerge to give rise to a dorsal root varies at each spinal segment; Bozkurt et al. [19] found that T_1_ segment contained the largest number of thoracic nerve rootlets, in contrast to the T_6_, T_7_, and T_10_ segments which contained the fewest. The central and dorsal rootlets form the segmental spinal nerve, converging in the TIF area. Two layers of pia mater, the arachnoid, and the dura mater surround the rootlets until they advance toward the IVF, then the pial and arachnoidian layers are fused with the dura mater of the thecal sac. A thin connective tissue sheath loosely surrounds the dorsal and ventral roots when they exit through separate perforations in the dura. It has been estimated that the mean foraminal width is 0.8 cm, while the widths of thoracic TP are between 1.2 and 1.3 cm [20,21]. With this premise, we speculated that the “*nerve root compartment*” of TIF is presumably 1.5 cm from the anterior limit of TP of vertebra. Thus, it can be filled with anesthetic solution by gently locating the needle 2 mm over and behind the angle between TP and SP, providing an effective block without the necessity of approaching the pleura from TPVS or the ES directly, and avoiding the attendant risks. The catheter placement should not exceed 1.5 cm from the tip of the needle, avoiding iatrogenic damages for the vulnerable structures of TIF. Shibata et al. described the costotransverse foramen block. This technique aims to posteriorly reach the TPVS via the costotransverse foramen [22]. They focused the US beam to identify the superior costotransverse ligament (SCTL) above the paravertebral space. The SCTL is a part of the interconnecting musculoaponeurotic system which forms the posterior boundary of TPVS together with the TP of vertebrae and the head and neck of the ribs [23]. The musculoaponeurotic system is also formed by the aponeurosis of the internal intercostal muscle (ICM) [24]. On the contrary, our procedure can be considered a modified approach to TPVB without penetrating into the TPVS through its boundaries. We aimed to place LA over and behind the transverse process (TP) of vertebra, by placing the needle tip along the superior limit of the vertebral pedicle until losing contact with the bone, to overcome the articular processes. While it is important to underline that, medially, TIF were found as TPVS boundaries; during our procedure, the needle tip was outside the TPVS (Figure 4).

Our study has limitations that should be underlined. The first limitation of our findings may be due to the width of TP of T_10_, which we estimated to be 1.1–1.2 cm. The second limitation may be due to the fact that the US beam cannot identify the TIF content and the needle tip behind the acoustic shadow from the TP and the vertebral articular processes (Figure 1). Nevertheless, iatrogenic damage was avoided by carefully advancing the needle tip along the vertebral pedicle no more than 2 mm until contact with bone ended. However, concerns about damaging a nerve root or blood vessel remain. It possible to speculate that the transforaminal ligaments and connective tissue sheath presumably protect the content of the TIF, maybe minimizing the risk for neural and vascular damages. We identified virtual dissection as a potentially useful option to allow the emulation of anatomical dissection as close as possible to reality, notwithstanding the physical absence of a corpse (Figure 4) [9]. Thus, we believe our findings might demonstrate the necessary safety of the procedure regarding this approach, as Figure 4 shows. However, cadaveric studies are requested to provide exact evidence of LA placement and spread and the exact location of the catheters.

In effect, the second-look ultrasound scan of TPVS was performed, documenting anechoic fluid in the TPVS at a level of T_8_ and presumably indicating the LA spread (Figure 3). This might indirectly demonstrate that the catheter tip location was in the correct position near the TIF area, although the US beam cannot clearly identify it. We designed a cadaveric study to demonstrate the anatomical implications of our findings. In addition, another limit of this report is that two cases or more should be described to demonstrate the efficacy and reproducibility of a new technique. However, we intend to perform clinical trials after the cadaver dissection study to confirm our speculations.

**Figure 5:**
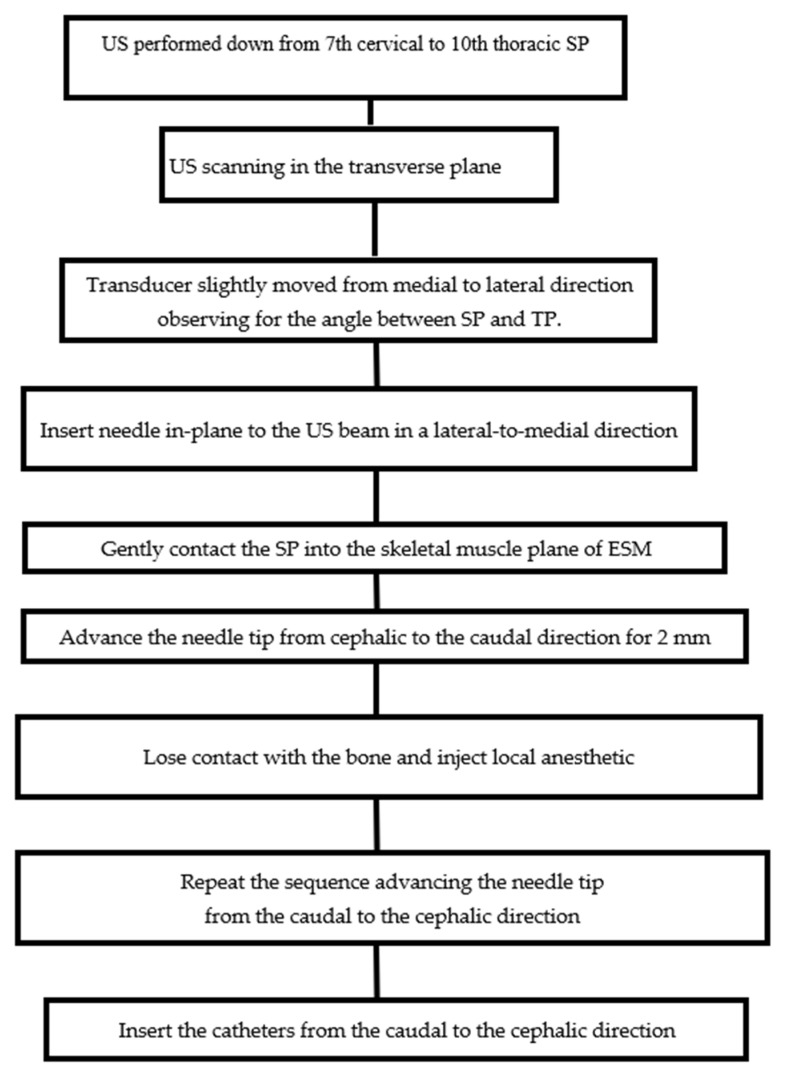
Flow diagram of the thoracic intervertebral foramen block. The main steps of the block are shown.

We are confident that our procedure is quite fast to perform. The US beam allows us to quickly identify the needle while the angle between SP and TP is reached. The needle tip is inserted into the skeletal muscle plane of the ESM by maintaining the contact with SP of vertebra. The US guidance and the contact with bone allows us to quickly place the needle and the catheters onto the anesthetic target, without delay for surgical operation, as the emergent laparotomy. The flow diagram depicts the main steps for a safe and quick procedure of the block, as shown in Figure 5.

## 4. Conclusions

In conclusion, based on our clinical observations, we are confident that use of the thoracic intervertebral foramen (TIF) block could be considered an effective alternative to thoracic paravertebral block (TPVB) and to other paraspinal anesthetic procedures, as an opioid-sparing strategy, for fast-track recovery after pediatric abdominal surgeries. Our case report provides important evidence for future randomized clinical trials.

## Data Availability

Not applicable.
